# Supporting the home care workforce across the Southern United States: impetus, exploration, and policy strategies

**DOI:** 10.1093/haschl/qxaf031

**Published:** 2025-03-12

**Authors:** Britt Sanderson, Sam Obbin, Kiera Williams, Stephen McCall, Matt Michaelson

**Affiliations:** Applied Research and Analytics, Department of Population Health, Altarum Institute, Arlington, VA 22201, United States; Health Economics and Policy, Department of Population Health, Altarum Institute, Arlington, VA 22201, United States; Applied Research and Analytics, Department of Population Health, Altarum Institute, Arlington, VA 22201, United States; Health Economics and Policy, Department of Population Health, Altarum Institute, Arlington, VA 22201, United States; Applied Research and Analytics, Department of Population Health, Altarum Institute, Arlington, VA 22201, United States

**Keywords:** home- and community-based services, American Rescue Plan Act, Southern state policy, home care workforce, direct care workforce, health disparities: policy strategies

## Abstract

Southern states face severe home care labor shortages due to low wages, poor working conditions, and limited career advancement opportunities, which are exacerbated by the region's historical labor policies and economic inequalities. This study analyzed workforce size in relation to the population of older adults likely to require paid home care services, subsequently contextualizing those results using a thematic analysis of state American Rescue Plan Act section 9817 spending plans to identify trends in proposed initiatives designed to strengthen the workforce across the region. Our findings highlight significant disparities in workforce availability, with more diverse areas with higher concentrations of Hispanic, immigrant, and low-income populations exhibiting higher workforce capacity compared with less diverse regions. We also found consensus across states on the inadequacy of direct care worker wages, demonstrated by the large number of proposed reimbursement rate increases included in the state spending plans.

## Introduction

More than 3.1 million people across the Southern United States have a chronic condition that impedes their ability to accomplish activities of daily living.^1^ (For the purposes of this brief, the “Southern United States” includes the District of Columbia, Alabama, Arkansas, Delaware, Florida, Georgia, Kentucky, Louisiana, Maryland, Mississippi, North Carolina, Oklahoma, South Carolina, Tennessee, Texas, Virginia, and West Virginia.) Southern states report the highest concentration of adults with self-care needs, but also a significant gap between the availability of home care workers and the demand for their services.^2^ The home care workforce, composed primarily of personal care aides, home health aides, and nursing assistants, play a vital role in assisting this population perform routine tasks, monitor their health, and ultimately remain in their homes.

Low wages, physical and emotional burnout, limited benefits, and minimal career advancement opportunities impede workforce recruitment and retention efforts.^2^ Across all industries, workers in the South are paid less and provided fewer benefits compared with their equivalents in the Northeast, West, and Midwest.^3^ The resultant workforce deficit exacerbates consumer burdens, such as limited respite for unpaid family caregivers and increased reliance on costly institutional care.^4^ Additionally, systemic discrimination and regressive labor policies, from Jim Crow to contemporary “right-to-work” laws, have entrenched poor working conditions for home care workers in the South.^5^

“Right-to-work” laws, which prevent labor unions from requiring membership or dues as a condition of employment, weaken collective bargaining power. This limits home care workers’ ability to advocate for higher wages, better benefits, and improved workplace protections.^6^ In 2023, the median annual salary for home health and personal care aides in across the region ranged from $20 800 in Louisiana to $37 010 in the District of Columbia, with wages for home care workers in 15 out of the 17 Southern states falling below the national median ($33 530).^7^

Over the last several years, the workforce deficit has become more prominent in the national discourse, driven largely by the coronavirus pandemic and the resulting decrease in the availability of home care workers.^8^ The growing interest in strengthening the workforce is reflected in a variety of recent state and federal initiatives. Preeminent among these, the American Rescue Plan Act (ARPA) extended federal funding to states to reinforce their existing health care infrastructure and respond to emerging threats.^9^

In response, Southern states proposed a variety of initiatives leveraging these funds, which represented the largest investment in Medicaid-funded home- and community-based services (HCBSs) in American history. However, tracking the implementation and efficacy of these proposals has been challenging due to varying reporting formats and inconsistent data availability.^10^ This analysis builds on the existing literature examining geographic variations in workforce size and adequacy across the region and contextualizes those results using a secondary thematic analysis of ARPA-funded activities proposed to address the workforce deficit.

## Data and methods

This mixed-methods study examines geographic variations in the home care workforce and explores how ARPA funding was proposed to address worker shortages in the Southern United States. By combining data from the American Community Survey (ACS) and ARPA Section 9817 State Spending Plans and Narratives, the analysis investigates workforce size, potential consumer needs, and state-level strategies to improve recruitment, retention, and working conditions for home care workers.

## Data sources and measures

- American Community Survey (ACS) 5-year (2018-2022) integrated public use microdata sample (IPUMS): A publicly available dataset from the University of Minnesota providing individual-level data on demographic, economic, and housing characteristics based on annual nationwide surveys conducted by the U.S. Census Bureau.
- Public Use Microdata Areas (PUMAs): Non-overlapping statistical geographic units defined by the U.S. Census Bureau, each containing at least 100 000 people. Public Use Microdata Areas facilitate detailed analysis while preserving respondent confidentiality. In this study, PUMAs are frequently referred to as “local areas.”
- American Rescue Plan Act Section 9817 State Spending Plans and Narratives: Documents submitted by states to the Centers for Medicare and Medicaid Services (CMS) detailing the use of increased federal medical assistance percentage funds under ARPA. These plans outline initiatives to enhance, expand, or strengthen HCBSs. States are required to submit quarterly spending plans and biannual narratives describing enhancement activities.
- Full-Time Equivalent (FTE) Home Care Worker Workforce Size: A measure of workforce size calculated by dividing the total weekly hours worked by home care workers in a PUMA by 40 h.
- Potential Home Care Consumer: Individuals aged 65 years or older with a physical or mental health condition lasting at least 6 months that limits their ability to perform personal care tasks such as bathing, dressing, or moving around the home.

## Methods

### Bivariate analysis and quartile analysis

Using data from the ACS 5-year (2018-2022) IPUMS, we conceptualize home care workforce abundance or shortage as the ratio of the number of home care workers available to the number of potential consumers. This ratio is a measure of the gap between the supply of and the demand for home care workers. To calculate this, we computed the ratio of FTE home care workers (labor supply) to the number of individuals who are likely to require home care assistance (demand).

Given that some home care workers are employed part-time, using the total count of home care workers could lead to an overestimation of the ratio, whereas using only full-time workers could lead to an underestimation. To address this, we introduced a variable termed “full-time equivalent home care workers” (hereafter referred to as workforce size), which was determined by dividing the total number of hours worked by all home care workers within each PUMA in Southern United States over the course of a week by 40 h.

PUMAs, hereafter referred to interchangeably as “local areas,” are non-overlapping statistical geographic areas that partition each state or equivalent entity into geographic areas containing no fewer than 100 000 people each. Potential consumers are defined as residents aged 65 years or older who experience any physical or mental health condition that has persisted for at least 6 months and impedes their ability to perform personal care tasks such as bathing, dressing, or moving around within the home.

We first conducted a bivariate analysis to examine variations in the gap between workforce supply and demand between rural and urban areas. Subsequently, we explored differences across states in the Southern region. We then assessed the variation across demographic groups by categorizing local areas into quartiles based on the proportion of the population that identified as Black/African American, Hispanic, immigrant, or living in poverty and computing the ratio for each quartile of each group. For example, to establish the immigrant category, we grouped the local areas into 4 equal quantiles to classify the areas with the highest percentage of immigrants to the areas with the lowest percentage of immigrants and determined workforce availability for each quartile.

**Figure 1.**
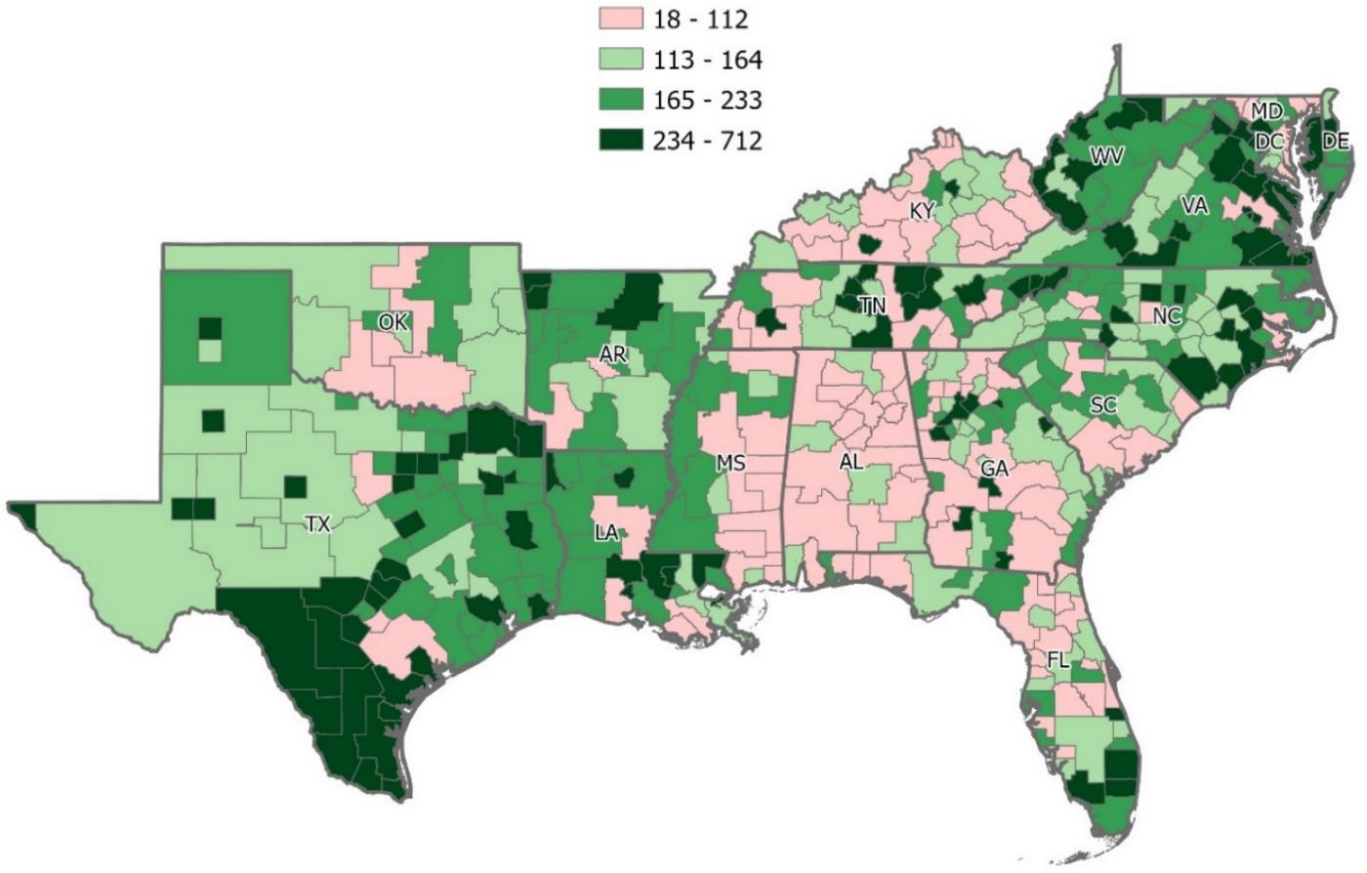
Number of home care workers per 1000 potential consumers based on percentage of consumers living in poverty in a given local area. Source: Authors analysis using data from the Integrated Public Use Microdata Sample (IPUMS); visualizations done by M.M., GISP, using ArcGIS. https://usa.ipums.org/usa-action/variables/METRO#description_section.

The quartile analyses provided a nuanced understanding of disparities in workforce availability across different geographic areas and demographic groups. By systematically examining variations in the supply of and demand for home care workers, this study sought to identify areas and groups with the greatest unmet needs. While research on geographic and demographic disparities in home care worker availability remains limited, this study builds upon findings from Chapman et al.,^2^ which explored the need for personal care aides in various regions. In this context, our research contributes to the existing knowledge base by evaluating workforce shortages across diverse groups and locations. Quantitative analyses were performed using Stata, while ArcGIS was employed for spatial mapping (see Figure 1).

### Thematic analysis

To assess the proposed utilization of federal funding under ARPA Section 9817, we conducted a thematic analysis of State Spending Plans and Narratives submitted to the CMS between January 2023 and September 2023.^11^ To begin, we collected the ARPA Section 9817 State Spending Plans and Narratives from 16 states and the District of Columbia. These included Alabama, Arkansas, Delaware, Florida, Georgia, Kentucky, Louisiana, Maryland, Mississippi, North Carolina, Oklahoma, South Carolina, Tennessee, Texas, Virginia, and West Virginia. Each spending plan was thoroughly reviewed to identify proposed initiatives, which were then extracted and compiled into a state-specific dataset.

Using the dataset of proposed initiatives for each of the selected states, we developed an initial codebook which reflected the observed patterns and priorities within the spending plans and our focus on state efforts to improve working conditions for home care workers identified in our early reviews. The initial codes were then iteratively refined through a second review to identify emerging themes. These themes were organized into a matrix, where themes and codes were listed down the *y* axis and states were listed across the *x* axis. This matrix served as the final database, where all initiatives were organized by state, theme, and code to identify the number of initiatives that fell under each.

Finally, the dataset was refined to include only initiatives explicitly targeting home care workers, excluding those related to consumer benefits, eligibility changes, and service line expansions. The final themes discussed in this analysis include “reimbursement rate increases,” “employee benefits,” “training and “professional development,” and “studies.”

### Limitations

Several limitations may impact the findings of our analysis. First, this analysis could not account for consumers’ reliance on family and unpaid caregivers and its effect on workforce demand. We may have observed fewer workers in a local area, or PUMA, because people relied more on family and unpaid caregivers and demand for their paid services was lower. Additionally, our analysis may have excluded individuals with multiple jobs. ACS respondents only report on their primary occupation, and home care workers frequently have second jobs.^12^

Finally, some paid family and friends might not self-identify as home care workers in census surveys when they are compensated through Medicaid consumer-directed programs, leading to an underestimate of workforce capacity. Despite these limitations, this work highlights the variations in the distribution and potential capacity of the home care workforce.

## Results

### Bivariate analysis and quartile analysis

The bivariate analysis showed that the ratio of the supply of home care workers to the potential demand by likely consumers was lower in non-metropolitan areas compared with metropolitan areas. While there were 287 home care workers available per 1000 likely consumers in metropolitan areas, there were 214 home care workers available per 1000 likely consumers in non-metropolitan areas. This finding is similar to Chapman et al.^2^ finding that, generally, there was greater shortage of home care workers in rural areas compared with non-rural areas.

Additionally, the quartile analysis uncovered significant regional variations in workforce capacity within the Southern United States (see Figure 1). Compared with other areas in the South, areas along the Texas–Mexico border and in areas closer to the mid-Atlantic region had higher potential capacity. In contrast, Alabama exhibited the greatest shortage of home care workers in the country, with only 97 workers per 1000 likely consumers. The District of Columbia, conversely, reported the highest ratio, with 570 workers per 1000 likely consumers, well above the regional average of 212 and median of 191 workers per 1000 likely consumers.

Of particular note, our analysis suggests that more impoverished areas and more diverse areas generally had greater numbers of home care workers per 1000 likely consumers. We found that the number of home care workers per 1000 likely consumers was higher in areas with the largest percentage of Hispanic or Latino consumers (260) compared with areas with the lowest percentage (184). Similarly, areas with the largest percentage of immigrants and people living in poverty exhibited ratios of 263 and 220 workers per 1000 likely consumers, respectively, whereas areas with the lowest percentage of immigrants and people living in poverty returned ratios of 176 and 197 workers per 1000 likely consumers. These findings help contextualize the comparatively high potential workforce size in areas along the US–Mexico border.

**Figure 2.**
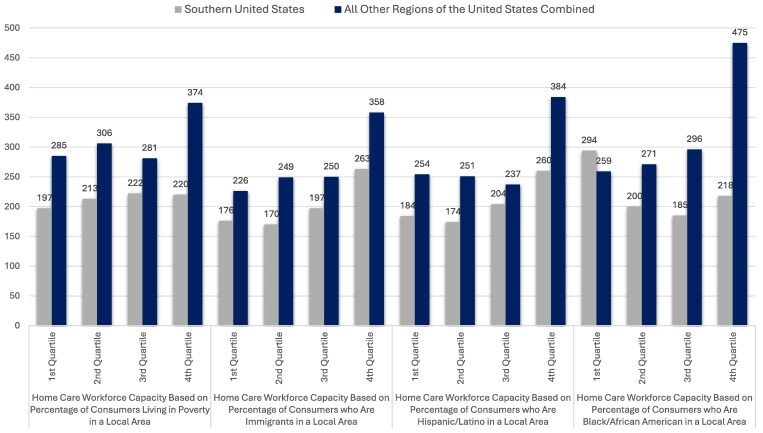
Home care workforce across local area quartiles by percentage of consumers, by demographic groups and region. Source: Authors analysis using data from the Integrated Public Use Microdata Sample (IPUMS). https://usa.ipums.org/.

Generally, the ratio of home care workers to likely consumers was significantly lower in the Southern United States compared with other regions in the country combined (see Figure 2). Based on our analysis, the local areas with the highest concentrations of Hispanic, immigrant, and low-income residents outside of the Southern United States returned potential ratios of 387, 358, and 374 potential home care workers per 1000 likely consumers, respectively, which exceed the ratios in the American South discussed in the paragraph above by notable margins.

### Thematic analysis

We found that each state surveyed proposed using ARPA 9817 funding to establish time-limited incentive payments for workers, longer-term reimbursement rate increases for Medicaid HCBS providers, or a combination of both. However, our analysis also identified substantial variation in the design and implementation of these initiatives across states.

Several states, including Alabama, Kentucky, Maryland, and Oklahoma, prioritized implementing permanent rate increases to strengthen the home- and community-based care workforce. In Kentucky, ARPA funds were used to support rate increases under Appendix K waivers, while the state conducted a comprehensive rate study. The Kentucky Department for Medicaid Services later adopted these increases permanently, establishing updated rates at a minimum of 70% of the benchmark levels identified in the study. Kentucky also approved a supplemental 50% rate increase for select services, including attendant care, personal care, and personal attendance, so long as the provider agency attests that at least 85% of the supplemental increase be used to increase wages or offer incentives to workers.^13^ Similarly, Alabama increased reimbursement rates for services provided by home health and direct service workers reimbursed under the state SAIL waiver, which provides services to disabled adults who would otherwise qualify for care in a nursing care facility.^14^

In contrast, several states directed the majority of their ARPA funding toward short-term financial support for care workers. Florida, South Carolina, and Arkansas offered one-time or temporary incentive payments to direct support providers, granting agencies flexibility to use these funds for hiring or longevity bonuses. Texas also prioritized incentive payments for care workers through reimbursement rate increases but coupled the increase with a mandate that at least 90% of the additional funds be allocated to 1-time financial compensation or paid leave for direct care workers. Similarly, Louisiana implemented time-limited rate increases with a stipulation that 70% of the additional funds be passed directly to direct support workers.

Other states pursued some combination of the two, adopting strategies that combined short-term incentives with long-term rate adjustments. For example, Virginia provided $1000 hazard payments to personal care attendants while simultaneously raising reimbursement rates by 12.5%-20%, which were subsequently made permanent.^15^ Likewise, North Carolina used ARPA funding to provide retainer payments, establish temporary and permanent rate increases, and implement a $15.00 minimum wage for direct care workers as part of its 2021 Appropriations Act.^16,17^

Georgia and the District of Columbia also adopted hybrid approaches. In addition to providing temporary payment enhancements to support the workforce, Georgia increased reimbursement rates for services provided under the state Independent Care Waiver and Elderly and Disabled Waiver Programs by 10% in an attempt to bolster workforce retention. The District of Columbia developed the Direct Care Service Provider Supplemental Payment program, aiming to increase direct care professionals’ wages to 117.6% of the District's minimum wage by FY 2025. This program also established a target wage rate, requiring providers reimbursed through Medicaid HCBS waivers to pay their workforce at least 10% above the higher of the District's minimum or living wage.

### Employee benefits

Although each state used the increased federal funding to improve reimbursement for HCBSs, the District of Columbia was the only state that explicitly included a proposal to extend employee benefits to home care workers under their Direct Care Worker Transportation Benefit proposal.

### Training and professional development

With the exception of Arkansas, Florida, Kentucky, Maryland, and Virginia, each state proposed strategies to enhance or develop workforce training programs. A few noteworthy initiatives include Alabama's proposed ARPA-funded training curriculum focused on human rights challenges within disability services and Delaware's LEAD program, which provides leadership training for care workers serving individuals with intellectual and developmental disabilities. In Texas, ARPA funds were used to establish an optional rate enhancement program incentivizing providers to offer wage increases tied to workers’ completion of competency-based trainings.

### Studies

Eleven states also reported that they have used, or plan to use, ARPA funds to conduct formal studies, convene task forces, or form commissions to guide future initiatives. In total, 7 states—including Alabama, Delaware, the District of Columbia, Georgia, Kentucky, Mississippi, and West Virginia—proposed commissioning a rate study specific to the state's HCBS providers. Seven states proposed using ARPA funding to evaluate the current state of their home care workforce. Alabama, the District of Columbia, and West Virginia proposed incorporating an evaluation of the state's care workforce as a component of their proposed rates studies, and Delaware, Georgia, Mississippi, and North Carolina proposed conducting evaluations of their workforce independent of a rate study.

## Discussion

Home care workers across the South face challenges that undermine the stability and quality of the workforce. The ratio of home care workers to home care consumers may serve as a valuable metric to guide efforts to address these challenges. Additionally, the observed variations in workforce supply across the region suggest that effective solutions will need to account for the unique conditions within each state. However, systemic labor issues—including low wages, limited access to employee benefits, and limited opportunities for collective bargaining—present significant obstacles that must be addressed to effectively resolve the workforce deficit.

Across all states surveyed, the insufficient workforce was repeatedly referenced as a significant barrier to care. The current demand for home care workers surpasses labor force supply, and this trend is expected to continue into the next decade.^4^ By 2035, workforce projections estimate that a total of 536 830 home health and personal care aides will be needed across the South to meet the demand for services, with the greatest demand for workers anticipated in North Carolina (50 940), Florida (111 180), and Texas (113 170).^18^

These projections and our analysis indicate that population size contributes to variations in workforce capacity. However, the concentration of lower income and immigrant populations also contribute to the observed differences between local areas. Immigrants make up 17% of the total labor force, but nearly one-third of the home care workforce.^19^ This trend may reflect immigrants’ greater willingness to take on home care roles and underscores the importance of supporting communities through policies that improve access to training, fair wages, and work authorization.

Our thematic review illustrated that many states are willing to develop strategies to improve working conditions given sufficient funding. Southern states proposed a variety of activities to increase wages for home care workers in their ARPA spending plans, although the material impact is difficult to determine at this time due to the limited number of evaluations conducted.^10^ Additionally, the sustainability of these initiatives remains uncertain. ARPA funding is time-limited, and states have the authority to discontinue special initiatives at their discretion.

Some states have, however, enacted permanent improvements through legislation. For example, Virginia recently became 1 of 2 states to codify mandates guaranteeing home care workers the ability to accrue paid sick leave.^20^ At the federal level, a recent CMS’ final rule, Ensuring Access to Medicaid Services, includes provisions which will require providers to allocate 80% of Medicaid payments for home health, homemaker, and personal care services toward their employee's compensation by FY30.^21^ Mandates compelling providers to allocate a set amount of funding toward wages are frequently referred to as “pass-through” requirements.^22^

States have the authority to adopt similar or more comprehensive pass-through requirements.^23^ For example, the District of Columbia has enacted permanent wage pass-through legislation that applies to HCBS providers, and Texas have enacted permanent wage pass-through laws that apply exclusively to direct care workers employed in facility-based settings, like nursing homes.^24^ States may also pursue adjunct policy mechanisms, like wage floors, to guarantee home care worker wages increase proportionately with reimbursement rate increases.

Wage floors stabilize compensation and may be used to minimize pay gaps between home care and other entry-level positions.^25^ Interested states may consider establishing or amending existing 1915(c) waivers or State Plan Amendments to establish a standard wage floor for home care workers reimbursed under state Medicaid HCBS waivers. States may also establish a wage floor legislatively by increasing reimbursement rates in their annual fiscal budget. Florida, for instance, enacted a $15.00 per hour wage floor for direct care workers, including home health workers, in their 2023 budget.^26^ Likewise, in 2021, Louisiana has established a $9.00 per hour wage floor for workers employed by Medicaid personal assistance service providers, who support adults with disabilities living in community settings.^27^

Policymakers may also consider mandating wage parity across settings.^28^ Wage parity laws are less common, but may be designed to be industry-specific, allowing them to be used to standardize wages and benefits across service types (eg, facility- and home-based services).^29^ The Texas Department of Health and Human Services has published recommendations to establish wage parity, noting that job seekers may be less likely to apply for roles reimbursed under the Community Living Assistance and Support Services waiver program due to pay discrepancies between waiver programs.^30^

To attract a broader pool of potential employees and support workforce mobility, states might also consider offering relocation stipends or expanding reciprocity agreements. In 2024, the District of Columbia introduced legislation that would allow direct care workers in Maryland and Virginia to work in the District, but the bill died in committee.^31^ Also in 2024, Kentucky introduced legislation to compel state agencies to explore establishing an interstate compact with neighboring states that would allow individuals needing care to receive covered waiver services in any of the compact states.^32^ However, these initiatives must be paired with efforts to guarantee a living wage to ensure that they remain effective over time.

## Conclusion

The Southern United States faces a critical shortage of home care workers, exacerbated by low wages and limited career advancement opportunities. Our analysis revealed variations in workforce size and capacity across the South, with metropolitan and more diverse areas exhibiting a higher supply of home care workers than rural regions. However, despite these pockets of higher capacity, overall workforce size remains inadequate, highlighting the ongoing need for policy intervention.

While ARPA provided some relief, there is concern about the sustainability of those investments. To build a stable and equitable workforce, Southern states may consider adopting a combination of permanent strategies, such as enacting wage floors and establishing pass-through requirements. By focusing on sustaining effective ARPA-funded workforce initiatives, Southern states can make progress toward ensuring that all individuals have access to the support they need to live independently and with dignity.

## Supplementary Material

qxaf031_Supplementary_Data
